# Centenary of University of Maryland

**Published:** 1907-06

**Authors:** 


### CENTENARY OF UNIVERSITY OF MARYLAND.

To fully appreciate the importance and significance of the event which the centennial exercises will commemorate we must take a wide range of vision over the conditions which existed 100 years ago and compare them with conditions which exist at the present time. At the time the Maryland Medical College opened her doors for the instruction of medical students Thomas Jefferson was President of the United States, and this country was engaged in a controversy with Great Britain, which subsequently led to the War of 1812-14. The population of the United States was less than 10,000,000, and this population was confined almost entirely to the States and Territories east of the Mississippi. This great nation was at that time a weak Power, struggling for existence and foreign recognition. At home the conspiracy of Aaron Burr and his famous trial at Richmond Va., resulting in a verdict of not guilty, had just come to a termination.

In Europe, Napoleon was at war with Prussia and Russia, the great battle of Eilau had been fought, and the Treaty of Tilsit, which attached Russia to France as an ally, had been signed by Napoleon and Alexander.

In 1807 Fulton exhibited a submarine torpedo and demonstrated the success of the first steamboat, to which he gave the name of "Cleremont," but to which the people of New York gave the name of "Fulton's Folly."

In 1807 the population of Baltimore was about 33,000. The northern limit of the city was about Franklin street. Greene street was the western limit and Barre street was the southern boundary. —Hospital Bulletin.

